# Human leukocyte antigen-DR expression might predict outcomes in severe sepsis, but diabetes mellitus cannot be ignored

**DOI:** 10.1186/s13054-017-1718-x

**Published:** 2017-06-19

**Authors:** Jun Li, Xianshi Zhou, Ye Ye, Guanghua Guanghua

**Affiliations:** Emergency Department, Guangdong Provincial Hospital of Chinese Medicine, affiliated to Guangzhou University of Chinese Medicine, 111 Dade Road, Yuexiu District, Guangzhou, 510120 China

We read with great interest, but also with some concern, the paper by Drewry et al. recently published in *Critical Care* [1]. In this observational study, the authors concluded that human leukocyte antigen-DR (HLA-DR) expression might be a more accurate predictor of mortality and acquisition of secondary infections than lipopolysaccharide-stimulated TNF-α production in critically ill patients.

The primary concern with the article is that it overlooks the effects that diabetes might exert on the outcomes. Notably, the ratio of comorbid diabetes was statistically higher in the survivors than the non-survivors (*P* = 0.034). Diabetes has close and complex relationship with sepsis—it can not only alter the immune response (including HLA-DR expression) [2, 3], but also influence the mortality rate and risk of infection in patients with sepsis [2]. In addition, hyperglycemia, insulin [2], and obesity [4] secondary to diabetes can also impact on both the immune system and critical outcomes. Thus, it did not seem persuasive that HLA-DR expression could completely predict outcomes without adjusting for the covariate of diabetes.

Similarly, the APACHE II and SOFA scores, which reflect the severity of the disease, were statistically lower in the survivors than the non-survivors (*P* < 0.001 and *P* = 0.009, respectively). However, in a prospective study on mHLA-DR [5], in which the severity level based on SAPS II or SOFA score was adjusted, the original statistical relationship between early mHLA-DR downregulation and outcomes in the whole population disappeared (*P* > 0.05). Thus, it is plausible and necessary to adjust APACHE II and SOFA scores for statistical analysis in this paper.

The two inaccuracies described above are examples of colinearity, which suggest that not only univariate but also multivariable analysis should be carried out in this study to exclude interactions between variables and make the conclusion more prudent and accurate.

In addition, we do not understand why the APACHE II and SOFA scores were calculated without the neurological component, which can be easily done as the study was described as prospective. Moreover, we suggest that receiver operating characteristics (ROC) curves for the outcomes and these two markers be drawn to compare and determine which is the better predictive marker and what is the best, most pragmatic threshold (i.e., maximized sum of sensitivity and specificity).

We appreciate the meaningful work of Drewry et al., which provides us with a new perspective on predictive indicators in severe sepsis, though the statistical methods in the study need some amendments.
